# ZDHHC22-mediated mTOR palmitoylation restrains breast cancer growth and endocrine therapy resistance

**DOI:** 10.7150/ijbs.70544

**Published:** 2022-04-04

**Authors:** Jiefeng Huang, Jie Li, Jun Tang, Yushen Wu, Fengsheng Dai, Ziying Yi, Yan Wang, Yunhai Li, Yue Wu, Guosheng Ren, Tingxiu Xiang

**Affiliations:** 1 Department of Breast and Thyroid Surgery, Shenzhen People's Hospital (The Second Clinical Medical College, Jinan University; The First Affiliated Hospital, Southern University of Science and Technology), Shenzhen 518020, Guangdong, China; 2 Department of General Surgery, Shenzhen People's Hospital, Shenzhen 518020, Guangdong, China; 3 Department of Dermatology, Shenzhen People's Hospital (The Second Clinical Medical College, Jinan University; The First Affiliated Hospital, Southern University of Science and Technology), Shenzhen 518020, Guangdong, China; 4 Chongqing Key Laboratory of Molecular Oncology and Epigenetics, The First Affiliated Hospital of Chongqing Medical University, Chongqing 400016, China; 5 Department of Endocrine and Breast Surgery, The First Affiliated Hospital of Chongqing Medical University, Chongqing 400016, China; 6 Chongqing Key Laboratory of Translational Research for Cancer Metastasis and Individualized Treatment, Chongqing University Cancer Hospital, Chongqing 400030, China

**Keywords:** Breast cancer, ZDHHC22, Palmitoylation, mTOR, Endocrine therapy resistance

## Abstract

Palmitoylation is essential for the classic hallmarks of cancers through regulating protein stability and protein-protein interactions. ZDHHC22, as a well-known member of palmitoyltrans-ferase family, its role has not been revealed in cancer. We found ZDHHC22 expression was significantly lower in estrogen receptor (ER) negative breast cancer (BrCa) tissues and cell lines, and its expression was positively corelated with the clinical prognosis of BrCa patients. The lower expression of ZDHHC22 might be caused by its promoter methylation. ZDHHC22 inhibited the proliferation capability of BrCa cells both in vitro and in vivo, depending on its encoding palmitoyltransferase activity. In terms of the mechanisms, ZDHHC22 reduced mTOR stability via palmitoylation and decreased the activation of the AKT signaling pathway. Furthermore, ectopic expression of ZDHHC22 could restore the sensitivity to tamoxifen therapy in MCF-7R cells. Collectively, ZDHHC22 may serve as a prognostic biomarker and therapeutic target, providing the theoretical foundation for exploring specific palmitoylation drugs targeted, especially for endocrine therapy-resistant BrCa patients.

## Background

Breast cancer (BrCa) is the most frequently occurring cancer and the leading cause of cancer death among females worldwide 1, 2. Although systemic therapy has markedly improved the prognosis, many BrCa patients inevitably develop recurrence and metastasis, which presents a large clinical difficulty 3. With proteomics technology development, S-palmitoylation (hereinafter simply palmitoylation) has attracted significant attention from researchers in cancer proliferation, metastasis, and chemotherapy resistance, including BrCa 4-6. As reversible post-translational modifications, palmitoylation involves the attachment of the 16-carbon atom fatty acid palmitate onto the modified protein, regulating protein localization, stability, trafficking, and protein-protein interactions 7, 8. Palmitoylation occurs at particular cysteine residues and dynamically regulates the assembly and partition of proteins due to the reversible thioester bond 9-11, and this modification has been found in more than 2,000 human proteins 12.

Previous studies have shown that protein palmitoylation is catalyzed by Asp-His-His-Cys (DHHC) -family in mammals 9, 13-15. The human DHHC family has 23 members, and its cysteine-rich domain contains a motif of DHHC, and this domain also binds to two Zn^2+^, so it is also called ZDHHC palmitoyltransferase 16. An increasing body of evidence links ZDHHC-mediated palmitoylation to tumorigenesis and therapy resistance in various cancers 5, 17-21. Chen et al. reported that ZDHHC5 was upregulated in glioma tissue, which correlated with p53 mutation. ZDHHC5-mediated palmitoylation of EZH2 promoted tumorigenicity of glioma stem cells 22. Another study demonstrated that silencing ZDHHC3 suppressed PD-L1 palmitoylation and expression enhanced T-cell immunity against the colorectal cancer cells 23. Furthermore, we previously identified that ZDHHC1 could suppress tumor growth and promote p53 signaling through p53 palmitoylation 24, 25. ZDHHC22 was previously found that correlate with virus replication in influenza 26, neuronal axonal growth in vivo 27, and palmitoylation regulation in calcium-activated potassium channels 28. However, to the best of our knowledge, few studies have reported the pathological functions of ZDHHC22 in cancers.

In this study, we found that the expression of ZDHHC22 was significantly associated with estrogen receptor (ER) status in BrCa due to its promoter methylation, and higher ZDHHC22 expression was associated with better relapse-free survival in BrCa patients. Gain/loss of function assays indicated ZDHHC22 could restrain BrCa cell proliferation and induce cell cycle arrest and apoptosis. These tumor-inhibiting effects are depending ZDHHC22 palmitoylation. Further, we investigated that ZDHHC22 reduced the stability of mTOR via regulating its palmitoylation, leading to a reduction in the activity of its downstream substrates. Finally, our results revealed that tamoxifen-resistant BrCa cells exhibiting mTOR hyperactivation could restore tamoxifen therapy sensitivity through ZDHHC22 overexpression. Altogether, these findings highlighted that ZDHHC22-mediated palmitoylation might provide a new direction for BrCa treatment.

## Methods

### Cell lines and tissue specimens

The human breast cancer cell lines are BT-549, MCF-7, MDA-MB-231, MDA-MB-468, SK-BR-3, T47D, YCC-B1, and ZR-75-1, normal mammary epithelial cell lines HMEC and MCF-10A were acquired from American Type Culture Collection. Breast cancer cell lines were cultured in PRMI-1640 (Gibco BRL, Karlsruhe, Germany) supplemented with 10% fetal bovine serum (FBS) (Invitrogen, Carlsbad, CA), 100 U/mL penicillin, and 100 μg/mL streptomycin (Gibco BRL, Karlsruhe, Germany) at 37°C with 5% CO_2_. The tamoxifen-resistant MCF-7 (MCF-7R) breast cancer cell was a gift from Dr. Lin^29^ (Shantou Affiliated Hospital of Sun Yat-Sen University) and was cultured in RPMI-1640 without phenol red and L-glutamine (Biological industries, Haemek, Israel) containing 10% charcoal-stripped FBS (Biological industries, Haemek, Israel) and 1 μM 4-hydroxytamoxifen (Sigma-Aldrich, MO, USA). Human breast cancer specimens and paired normal tissues were collected at the First Affiliated Hospital of Chongqing Medical University. Each patient provided informed consent. The acquisition of human specimens was authorized by the Institutional Ethics Committee of the First Affiliated Hospital of Chongqing Medical University.

### Reverse transcription PCR (RT-PCR) and Quantitative real-time PCR

Total RNA was isolated from cell and tissue samples using TRIzol^®^ (Invitrogen, Carlsbad, CA). Reverse transcription was performed using the Promega GoScript™ reverse transcriptase (Promega, Madison, WI). RT-PCR was performed using Go-Taq (Promega, Madison, WI, USA) and 2% agarose gels. β-actin was used as a reference control. Quantitative reverse transcription PCR (qRT-PCR) was performed using SYBR Green (Thermo Fisher, Waltham, MA, USA) and 7500 Real-Time PCR System (Life Technologies, Thermo Fisher, USA). GAPDH was used as a reference control. The primer sequences are listed in Table S1.

### Bisulfite conversion and methylation-specific PCR (MSP)

Genomic DNA was obtained from cells and tissues. Bisulfite conversion of DNA samples was conducted as previously reported 30. The primers used for MSP are listed in table S2. MSP was performed using AmpliTaq^®^-Gold DNA polymerase (Applied Biosystems, US), and the results were analyzed as characterized previously described 31.

### Construction of plasmids and stable cell lines

The ZDHHC22 full-length gene with or without C111A mutation was inserted into a pCMV6-Entry plasmid to generate ZDHHC22 and ZDHHC22 mutation expression vectors. The recombinant DNA plasmid was screened and sequenced. pCMV6-Entry, pCMV6-ZDHHC22, and pCMV6-ZDHHC22-Mut were transfected into BT-549, SK-BR-3, and YCC-B1 cells using Lipofectamine 2000 (Invitrogen, CA, USA) according to the manufacturer's protocol. After 48 hours, cells were treated with G418 (200 μg/mL for BT-549, 500 μg/mL for SK-BR-3, and 400 μg/mL for YCC-B1). Stable overexpression of ZDHHC22 and ZDHHC22-Mut was confirmed by western blot and RT-PCR before other assays.

### Cell proliferation assay

Cells were seeded in a 96-well plate at a density of 2,000 cells per well. After 24, 48, and 72 hours, the Cell Counting Kit 8 (CCK-8, Beyotime, Jiangsu, China) was used to measure cell viability according to the manufacturer's protocol. At the indicated time points, optical density (OD) values were measured using a microplate reader (Infinite 200 PRO, TECAN, Männedorf, Switzerland) at a wavelength of 450 nm. Each assay was repeated three times independently.

### Colony formation assay

Cells were seeded into 6-well plates at 800 cells per well and incubated for 14 days. Cells were fixed with 4% paraformaldehyde and stained crystal violet (C0121, Beyotime). Visible colonies were counted, and experiments were repeated three times.

### Flow cytometry analysis of cell cycle and apoptosis

Cells were transfected with pCMV6-Entry, pCMV6-ZDHHC22, or pCMV6-ZDHHC22-(C111A) for the cell cycle assay. After 48 hours, cells were collected and fixed in 70% cold ethanol overnight. Fixed cells were incubated with 100 μL RNase A (0.1 mg/mL) at 37°C for 30 min and then stained with 5 μL propidium iodide (PI, Sigma-Aldrich, St. Louis, MO, USA) at room temperature for another 30 min in the dark. Data were collected using a FACSCalibur flow cytometer (BD Biosciences, Franklin Lakes, NJ, US) and analyzed using CellQuest™ software (BD Biosciences). For the apoptosis assay, transfected cells were harvested. After incubation with APC annexin V (BioLegend, San Diego, CA) and PI (BioLegend, San Diego, CA) at room temperature for 30 min, the proportion of apoptotic cells was analyzed with the FACSCalibur flow cytometer. Experiments were repeated three times independently.

### Western blot

Western blot was performed as previously described 32 with the following primary antibodies: anti-mTOR (1:100, sc-517464, Santa Cruz), anti-mTOR (1:1,000, #2972, Cell Signaling Technology), anti-phospho-IGF-1 Receptor (1:1000, #3021, Cell Signaling Technology), anti-phospho-HER-2(1:100, sc-81507, Santa Cruz), anti-Protor1 (1:100, sc-390496, Santa Cruz), anti-DEPTOR (1:100, sc-398169, Santa Cruz), anti-PDK1 (1:200, sc-293160, Santa Cruz), anti-PI3K p85α (1:100, sc-1673, Santa Cruz), anti-phospho-PI3K (1:100, sc-12929, Santa Cruz), anti-phospho-PI3K (1:1000, #4228, Cell Signaling Technology), anti-AKT (1:1,000, #4691T, Cell Signaling Technology), anti-phospho-AKT(S473) (1:1,000, #4060T, Cell Signaling Technology), anti-PHLPP2 (1:1,000, ab71973, Abcam), anti-phospho-AKT (T308) (1:100, sc-271966, Santa Cruz), anti-phospho-BAD (1:1000, #5284, Cell Signaling Technology), anti-phospho- GSK3β(Ser9) (1:1,000, #9323, Cell Signaling Technology), anti-phospho-FoxO1(Ser256) (1:1000, #9461T, Cell Signaling Technology), anti-phospho-4EBP1 (1:200, sc-293124, Santa Cruz), anti-phospho-p70S6K (1:200, sc-8416, Santa Cruz), anti-GAPDH (1:1,000, sc-47724, Santa Cruz), and anti-Myc-Tag (1:1,000, #2276s, Cell Signaling Technology).

### Immunofluorescence

Cells were seeded on coverslips and then transfected with pCMV6-Entry, pCMV6-ZDHHC22, or pCMV6-ZDHHC22-(C111A). After 48 hours, cells were fixed with 4% paraformaldehyde for 20 min and then permeabilized with 0.5% Triton X-100 for 10 min. After blocking with blocking buffer, cells were incubated with primary antibodies overnight at 4°C and then incubated with secondary antibodies for 1 hour at 37°C. DAPI (Roche, Palo Alto, CA, USA) was used for DNA counterstaining. Photomicrographs were acquired with a confocal laser scanning microscope (Leica, Hilden, Germany). The following antibodies were used for immunofluorescence: anti-Flag (1:400, #14793, Cell Signaling Technology), anti-mTOR (1:200, #2983, Cell Signaling Technology), anti-phospho-AKT(S473) (1:200, #4060T, Cell Signaling Technology), anti-PHLPP2 (1:40, ab71973, Abcam), ER-Tracker Red (1:2000, C1041, Beyotime), CoraLite488 (1:200, SA00013-1, Proteintech), and CoraLite594 (1:200, SA00013-4, Proteintech).

### Immunoprecipitation and acyl-biotin exchange assay

The acyl-biotin exchange (ABE) assay was performed to detect protein palmitoylation as previously described by Brigidi et al. 33. First, the target protein was purified with a specific antibody and immobilized on magnetic beads. The purified target protein was then treated with N-ethylmaleimide to irreversibly block the free thiol groups combined with unmodified cysteine residues. Secondly, the protein was treated with hydroxylamine, which led to specific cleavage of palmitoylated cysteine residues and exposure of a free palmitoylated thiol group. The palmitoylated thiol group was subjected to specific biotinylation with biotin-BMCC. Finally, the biotinylated target protein was eluted for western blot analysis with a streptavidin-horseradish peroxidase (HRP) antibody to quantify palmitoylation of the purified protein.

### In vivo tumor xenograft model

Animal experiments were conducted according to guidelines approved by the Ethics Committee of The First Affiliated Hospital of Chongqing Medical University. BT-549 cells stably transfected with ZDHHC22, ZDHHC22-Mut, or empty vector (1 × 10^8^ cells) were injected subcutaneously into the mammary fat pads of female nude mice (aged 4-6 weeks, weighing 18-22 grams, five mice per group). Xenograft size was measured every 2 days by a Vernier caliper and then calculated using the following formula: tumor volume = 0.5 × length × width^2^. After 15 days of injection, the mice were sacrificed, and the xenografts were isolated and measured. Xenografts were fixed in formalin for 48 hours and dehydrated overnight. After embedding into paraffin, the xenografts were sliced into 4 μm sections for immunohistochemistry (IHC) staining or immunofluorescence.

### Immunohistochemistry (IHC) staining and TUNEL assays

IHC staining was conducted as previously published protocol with the following primary antibodies: anti-Ki67 (1:200, #9449, Cell Signaling Technology). TUNEL detection kit (Beyotime Institute of Biotechnology, Nanjing, China) was subjected to TUNEL assays, and the results were acquired with a confocal laser scanning microscope (Leica, Hilden, Germany).

### Gene Expression Omnibus dataset analysis

Gene expression data of ZDHHC22 was acquired from the Gene Expression Omnibus (GEO) (GEO: GSE65194 and GSE21653, which contain 152 and 266 breast cancer cases, respectively; https://www.ncbi.nlm.nih.gov/geo/). Patients were classified into the ZDHHC22-high and ZDHHC22-low groups according to the median ZDHHC22 expression level (229805_at). Gene set enrichment analysis (GSEA) was completed using Broad Institute GSEA software 4.0 as previously described 34. Genes were considered significantly enriched if the normal *P*-value was below 0.05 and the false discovery rate was below 0.25.

### Statistical analysis

Statistical analyses were performed with GraphPad Prism 7.0 and IBM SPSS 22.0 software. All results are typical of at least three independent in vitro assays. The two-tailed Student's t-test, the chi-square test, and Fisher's exact test were used to determine assay results. If the *P*-value was below 0.05, results were considered statistically significant.

## Results

### The ZDHHC22 expression level in BrCa is associated with estrogen receptors due to promoter methylation

**Figure 1:**
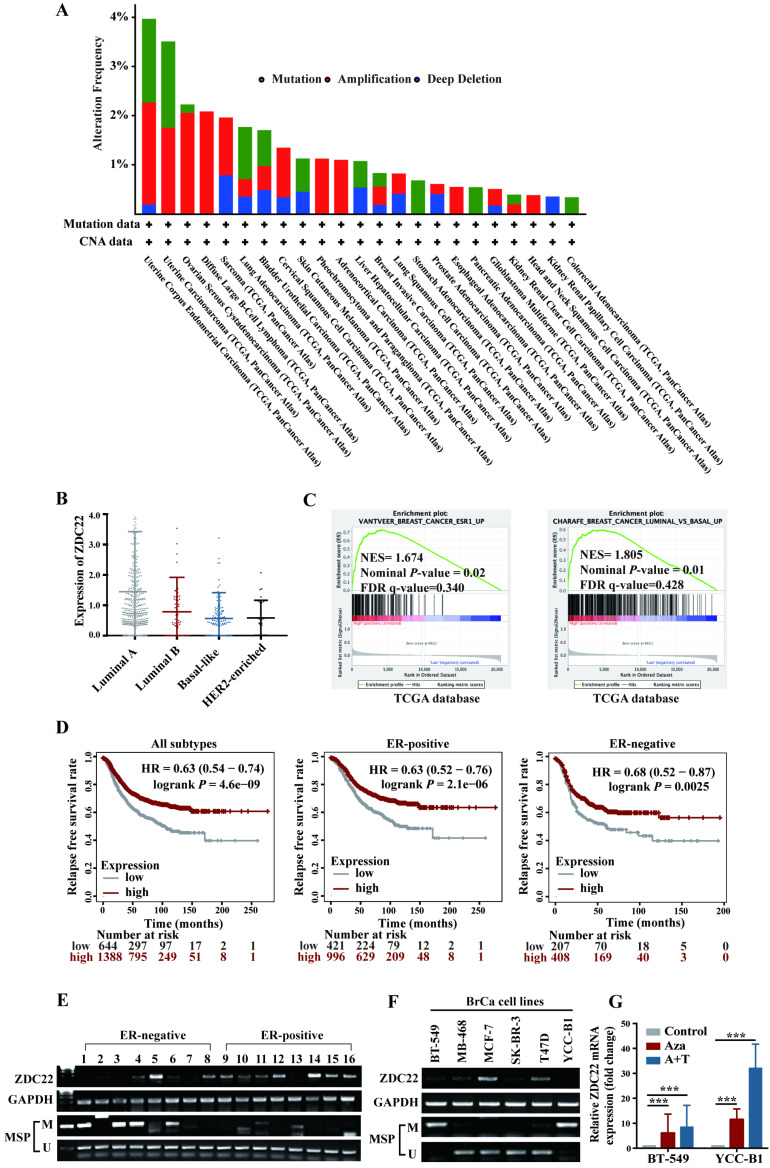
** Expression and methylation of ZDHHC22 in breast cancer. A.** Genetic alternations frequencies of ZDHHC22 in various carcinoma types. Green, genetic mutations; red, gene amplifications; blue, deep deletions. **B.** The ZDHHC22 mRNA expression in different breast cancer subtypes from the TCGA database. For purposes of exposition, ZDHHC22 is abbreviated as ZDC22 in the Figures.** C.** GSEA analysis of ZDHHC22 in BrCa using TCGA database. **D.** Kaplan-Meier (www.kmplot.com/analysis/) analysis of the correlation between ZDHHC22 expression and relapse-free survival of BrCa patients. **E-F.** ZDHHC22 mRNA expression in BrCa tissues and cell lines was examined by RT-PCR, and the methylation state of the ZDHHC22 promoter was detected by methylation-specific PCR (MSP). **G.** The expression of ZDHHC22 was detected by qRT-PCR after demethylation treatment with Aza and TSA in BrCa cell lines. Values represent mean ± SEM. ***P < 0.001.

Analysis of The Cancer Genome Atlas (TCGA) Pan-Cancer studies identified that alterations of ZDHHC22 like mutations, deletions, and amplifications occur in cancers of uterus, endometrium, ovarian, and breast (Fig. 1A). Besides, we analyzed the relationship between ZDHHC22 expression and clinicopathologic features in BrCa using the TCGA dataset. High ZDHHC22 expression was significantly associated with estrogen receptor (ER), progesterone receptor (PR), and human epidermal growth factor receptor 2 (HER2) status (Table 1). The ZDHHC22 (abbreviated as ZDC22 in the Figures) expression was decreased in HER2-enriched and basal-like breast carcinoma, which had been verified as "more aggressive" subtypes, compared with luminal-like subtypes (Fig. 1B). Gene Set Enrichment Analysis (GSEA) revealed that ZDHHC22 is significantly associated with ER and luminal-like subtypes (Fig. 1C). Kaplan-Meier plots (www.kmplot.com/analysis/), which included 2032 cases from the GEO and TCGA databases, revealed that higher ZDHHC22 expression was associated with better relapse-free survival in BrCa patients, especially in ER-positive BrCa patients (Fig. 1D).

We next examined the ZDHHC22 expression in 16 BrCa tissues and found that ZDHHC22 expression was significantly higher in ER-positive breast tumors (Fig. 1E). Within different BrCa cell lines, RT-PCR (Fig. 1F) also showed that ZDHHC22 expression was downregulated in most ER-negative BrCa cell lines (BT-549, MB-468, SK-BR-3, and YCC-B1) compared with ER-positive BrCa lines (MCF-7 and T47D). These results indicated that ZDHHC22 might be a valuable biomarker in BrCa tumorigenesis and progression.

CpG methylation is a crucial mechanism for gene expression regulation 35. Sequence analysis of ZDHHC22 uncovered a CpG island in the ZDHHC22 promoter. Thus, the promoter methylation status in the same 16 BrCa tissue samples and cell lines was detected using methylation-specific PCR (MSP) assay (Fig. 1E, F). The results observed that, in most cases, ZDHHC22 expression levels were inversely correlated with its promoter methylation status. Besides, the hypermethylation status of ZDHHC22 was observed in ER-negative tumors and cell lines. We next maximized the sample size to 64 BrCa tissue samples and found that the methylation status of ZDHHC22 was significantly associated with ER status (Table 2 and Fig S1A). Lastly, two hypermethylated cell lines, BT-549 and YCC-B1, were chosen for demethylation treatment. We observed that ZDHHC22 expression was efficiently restored (Fig. 1G), which validated that promoter methylation contributes to the expression of ZDHHC22 and its differential expression in various subtypes of BrCa.

### ZDHHC22 restrains BrCa cell proliferation through cell cycle arrest, and apoptosis depends on its enzymatic activity

**Figure 2:**
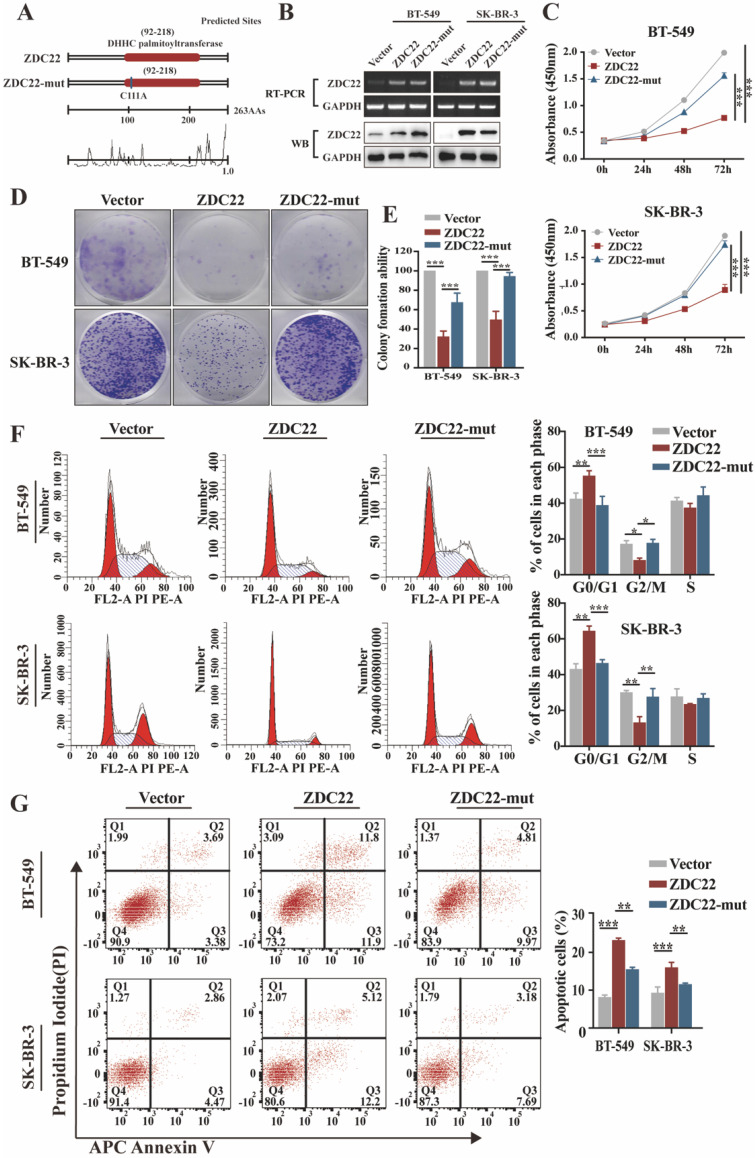
** Ectopic ZDHHC22 inhibits breast cancer cell proliferation, while ZDHHC22-(C111A) mutation abolished these effects. A.** Prediction and mutation of the active site in ZDHHC22 DNA sequence. The mutation site is shown as blue boxes. **B.** Validation of ZDHHC22 and ZDHHC22-(C111A) mRNA and protein expression by qRT-PCR and western blot analyses. **C-E.** Cell vitality was detected with CCK-8 assay and colony formation assay after transfection with Vector, ZDHHC22, and ZDHHC22-(C111A) plasmids in BT-549 and SK-BR-3 cells. **F-G.** Cell cycle and cell apoptosis in BT-549 and SK-BR-3 cells at 48 hours post-transfection of Vector, ZDHHC22, and ZDHHC22-(C111A) plasmids were examined by flow cytometry analysis of PI staining and Annexin V-APC/PI staining, respectively. Values represent mean ± SEM. from three independent experiments, **P < 0.01. ***P < 0.001. ZDC22: ZDHHC22, ZDC22-mut: ZDHHC22-(C111A) mutation.

Palmitoylation is catalyzed through the DHHC motif of palmitoyl S-acyltransferases 5, 9. To investigate whether the effects of ZDHHC22 on BrCa progression depend on its enzymatic activity, we created a point mutation (C111A) in the DHHC motif of ZDHHC22 (abbreviated as ZDC22-mut in the Figures) (Fig. 2A). Next, ER-negative BrCa cell lines BT-549, SK-BR-3, and YCC-B1 were stably transfected with ZDHHC22, ZDHHC22-C111A mutation or control plasmids to research the function of ZDHHC22. Restoration of ZDHHC22 was confirmed by RT-PCR and western blot (Fig. 2B and Fig.S1B). As the results of CCK-8 and colony formation assays, ZDHHC22-overexpression suppressed proliferation of BrCa cells (Fig. 2C-E and Fig. S1C-D), while the cell viability in the C111A mutation group was close to the control group, suggesting that the ZDHHC22 suppressive effects on BrCa proliferation probably depend on its enzymatic activity. Cell cycle analysis revealed that ZDHHC22 significantly increased the proportion of cells in the G0/G1 phase while decreasing the proportion of cells in the G2/M phase (Fig. 2F and Fig. S1E). Annexin V/PI staining and Flow cytometry analysis also revealed that the proportion of apoptotic cells was increased in the ZDHHC22 group compared with the control and C111A groups (Fig. 2G and Fig. S1F).

**Figure 3:**
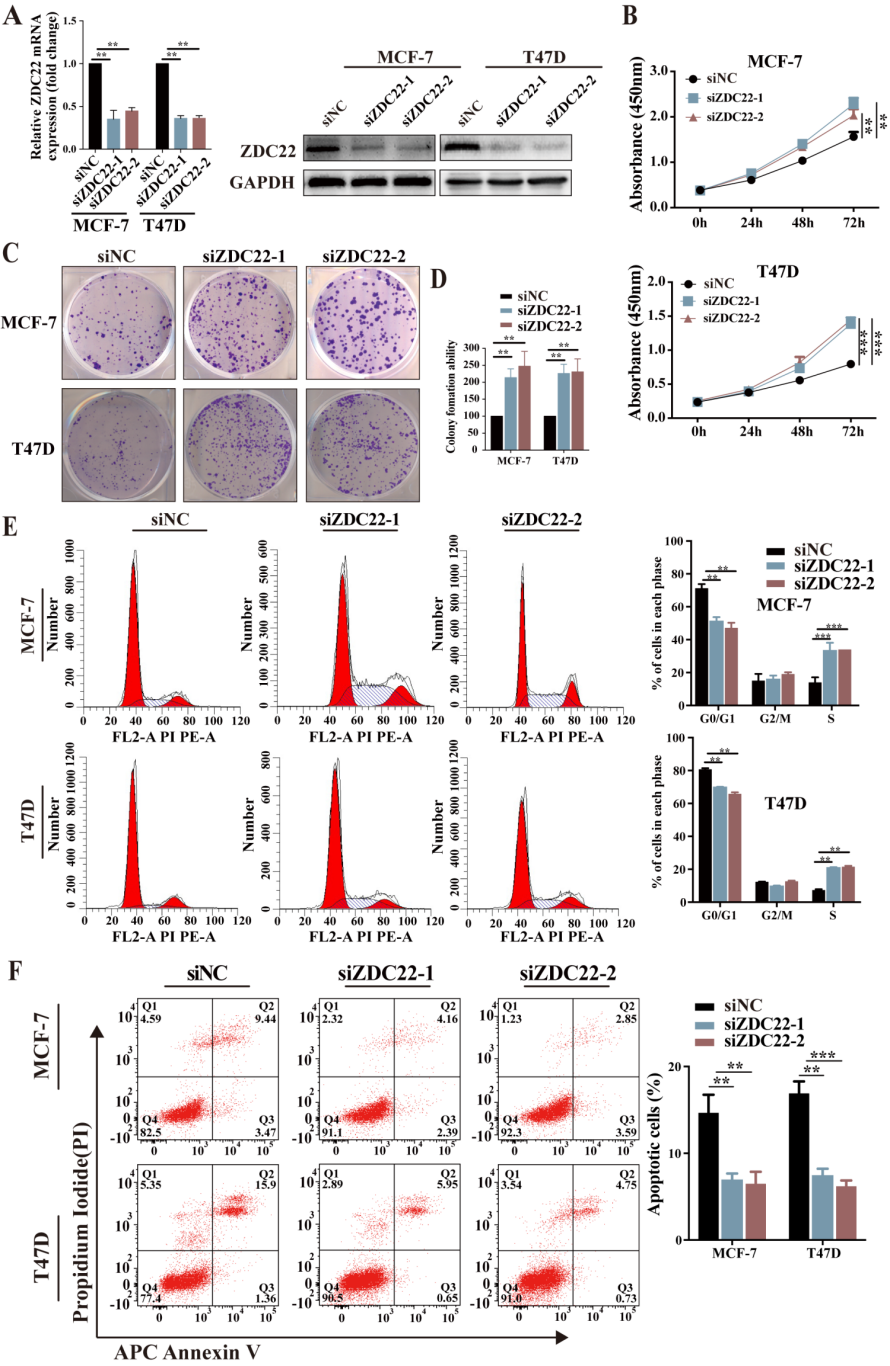
** Knockdown of ZDHHC22 promotes BrCa cell proliferation, relieves G0/G1 arrest, and inhibits apoptosis. A.** ZDHHC22 siRNA was transfected into MCF-7 and T47D cells, and the expression levels of ZDHHC22 were detected by qRT-PCR and western blot. **B-C.** MCF-7 and T47D cells were transfected with ZDHHC22 siRNAs, cell proliferation capacity was detected at the indicated time points by CCK8 assays and colony formation. **E-F.** Cell cycle and the percentage of apoptotic cells were analyzed by flow cytometry after the knockdown of ZDHHC22 in MCF-7 and T47D cells. Data are presented as the mean ± SEM. of three independent experiments. **P < 0.01. ***P < 0.001. ZDC22: ZDHHC22.

To further assess the suppression function of ZDHHC22 on ER-positive BrCa cell lines, loss-of-function assays were performed in ER-positive BrCa cell lines MCF-7 and T47D. After MCF-7 and T47D were transfected with two siRNAs of ZDHHC22, knockdown efficiency was detected by qRT-PCR and western blotting (Fig.3A). CCK-8 and colony formation assays revealed that knockdown of ZDHHC22 promoted the proliferation of BrCa cells (Fig. 3B-D). Besides, we found that the depletion of ZDHHC22 significantly decreased the rate of cells in the G0/G1 phase while increasing the rate of cells in the S phase (Fig. 3E). Flow cytometry analysis also showed that apoptotic cell proportion declined while the knockdown of ZDHHC22 (Fig. 3F). These results indicated that ZDHHC22 suppresses BrCa cell growth through cell cycle arrest and apoptosis. These inhibitory effects depend on the palmitoyltransferase activity of ZDHHC22 protein.

### ZDHHC22 reduces activation of AKT pathway

**Figure 4:**
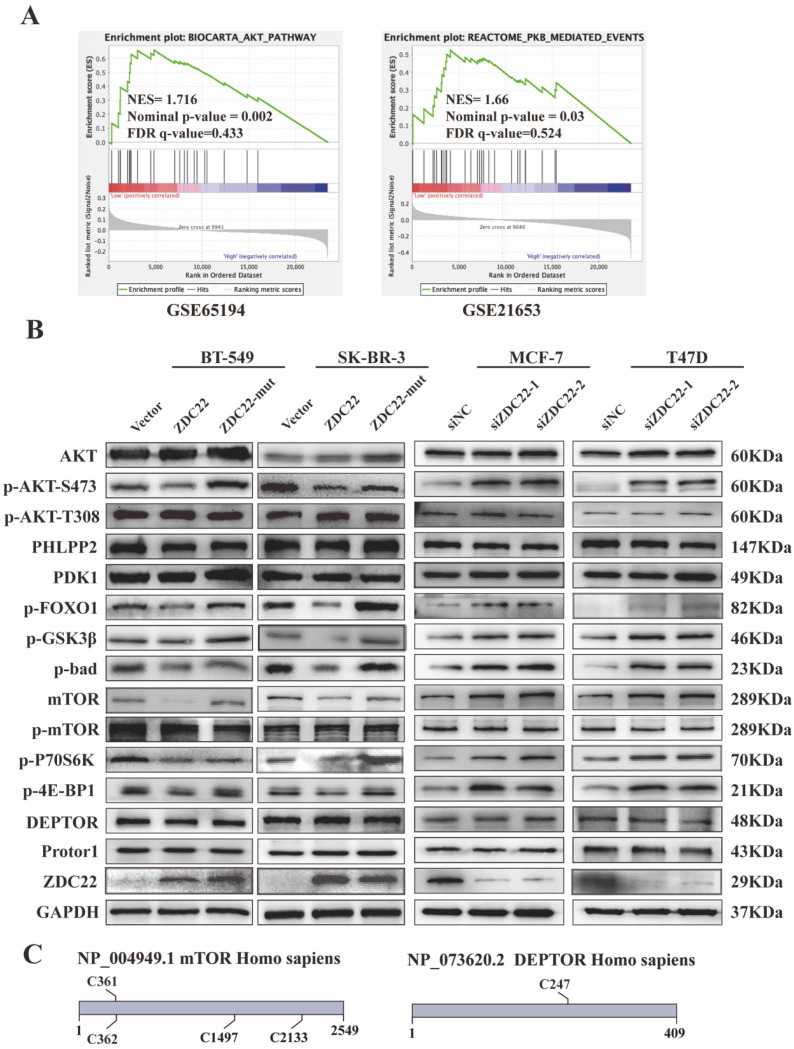
** ZDHHC22 reduces AKT pathway activation. A.** Gene set enrichment analysis (GSEA) based on microarray datasets GSE65194 and GSE21653. **B.** The western blotting assay was used to detect the expression of AKT signaling pathway-related molecules. **C.** Predicted cysteine residues on mTOR and DEPTOR susceptible to S-palmitoylation using palmitoylation site prediction tool CSS-Plam 4.0. ZDC22:ZDHHC22, ZDC22-mut: ZDHHC22-(C111A) mutation.

To further research the molecular mechanisms by which ZDHHC22 inhibits breast cancer, GSEA analysis was performed with data from the GEO database (datasets GSE65194 and GSE21653). The results indicated that the AKT/protein kinase B (PKB) pathway and PKB-mediated events were significantly correlated with ZDHHC22 (Fig. 4A). It has been verified that activating the AKT signaling pathway facilitates cell growth and survival. Thus, we hypothesized that ZDHHC22 overexpression might suppress tumor growth by reducing the activation of the AKT pathway. We next assessed a subset of proteins belonging to the AKT pathway by western blots. The ZDHHC22 overexpression led to decreased phosphorylation of AKT at Ser-473 (p-AKT(Ser-473)) compared with the control and C111A groups (Fig. 4B). In addition, the downstream substrates of p-AKT(Ser-473), phosphorylation of FOXO1, GSK3β, and Bad, were also decreased in the ZDHHC22 group. In contrast, phosphorylation at Thr-308 (p-AKT(Thr-308)) and total AKT protein levels remained unchanged. PDK1, which directly phosphorylates AKT at Thr-308 36, 37, remained unchanged in the ZDHHC22 group (Fig. 4B). These results indicated that ZDHHC22 decreased the p-AKT(Ser-473) rather than p-AKT(Thr-308).

### The mTOR protein is the substrate of ZDHHC22

The suppressive function of ZDHHC22, which we described previously, appeared during gain-of-function assays and was abolished after C111A mutation in the enzymatic motif of ZDHHC22. As palmitoyltransferases, the effects of ZDHHC22 on BrCa progression depend on its enzymatic activity. Thus, we concentrated on the proteins that regulate the AKT signal and could be modified by palmitoylation.

The phosphorylation of AKT at Ser-473 is regulated by two main phosphatases/complexes, PHLPP2, a phosphatase that directly dephosphorylates AKT(Ser-473) 38, and mTORC2, a kinase complex that phosphorylates AKT (Ser-473) 39. As shown in Fig. 4B and S1G, PHLPP2 showed similar expression levels in the three groups confirmed by western blot and immunofluorescence assays.

**Figure 5:**
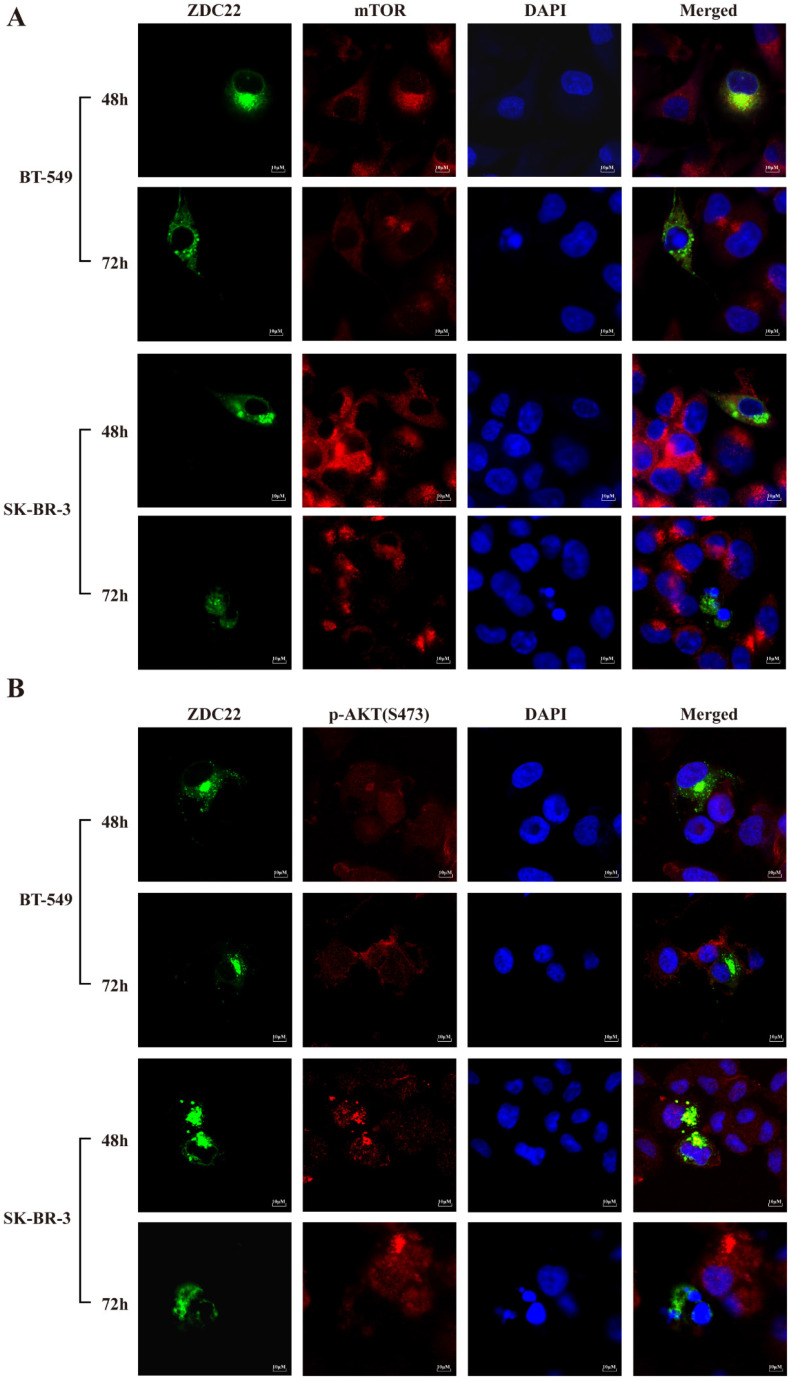
** ZDHHC22 reduces mTOR and phosphorylation of AKT at Ser-473 in a time-dependent manner. A-B.** Immunofluorescence assays were used to detect the protein expression and co-localization of mTOR (A) and phosphorylation of AKT at Ser-473 (B) at the indicated time in BT-549 and SK-BR-3 cells transfected with ZDHHC22 plasmids. ZDC22:ZDHHC22.

The mTORC2 consists of six components: mTOR, mLST8, Rictor, DEPTOR, mSin1, and Protor1/2 40. We predicted that only mTOR and DEPTOR might be regulated by S-palmitoylation using SwissPalm 12 and TermiNator 41 online tools (Fig. 4C and Table S3). As shown in Fig. 4B, the expression of total mTOR was decreased by ZDHHC22 overexpression, while the expression of DEPTOR remained unchanged. Immunofluorescence assays also revealed that ZDHHC22 decreased the expression of mTOR and promoted the generation of apoptotic bodies after 72 hours of transfection, while C111A groups remained unchanged (Fig. 5A and Fig. S2A). These changes during different time points are in line with the decrease of p-AKT(Ser-473) (Fig. 5B) and cell apoptosis after ZDHHC22 overexpression. Thus, we supposed that it might be the palmitoylation of mTOR by ZDHHC22 that led to the decrease of mTOR level, then restricted the kinase activity of mTORC2, and resulted in the decreased p-AKT(Ser-473) eventually.

Besides, the phosphorylation level of pS6K kinase and 4E-BP1, the downstream substrates of mTORC1, were also decreased in the ZDHHC22 group (Fig. 4B). Taken together, we hypothesized that ZDHHC22 might decrease both the activation of mTORC2 and mTORC1 through attenuating mTOR expression via palmitoylation.

### ZDHHC22 attenuates mTOR stability through mTOR palmitoylation

**Figure 6:**
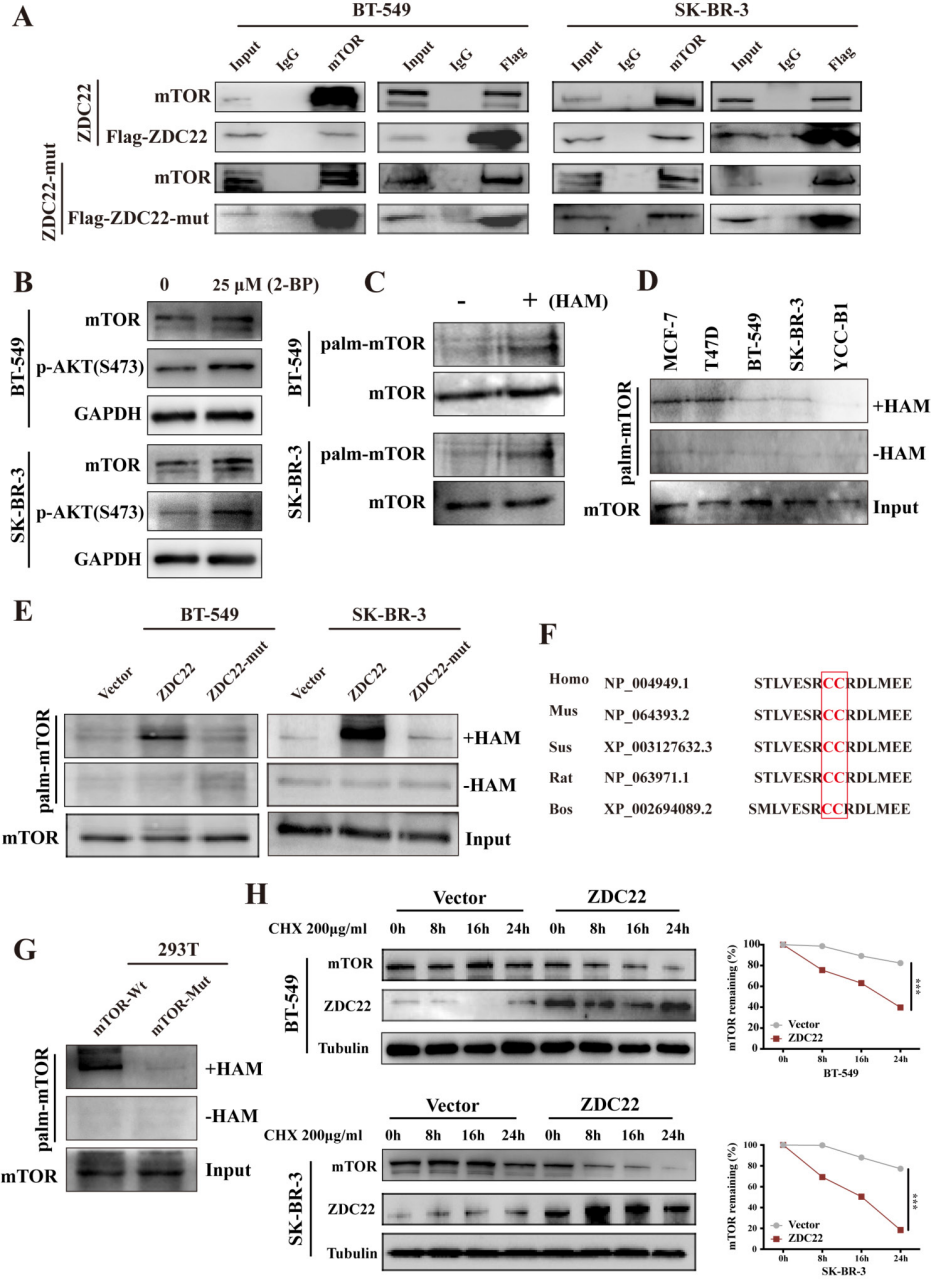
** ZDHHC22 attenuates mTOR stability via palmitoylation. A.** Stable expression of ZDHHC22^Flag^ and ZDHHC22-C111A^Flag^ in BT-549 and SK-BR-3 cell, the indicated proteins were detected by western blot after co-immunoprecipitation with Flag antibodies or mTOR antibodies. **B.** Expression of mTOR and p-AKT(Ser-473) in BT-549 and SK-BR-3 cells was elevated after being treated with 25μM 2-bromopalmitate for 12h by using western blot. **C.** Palmitoylation of mTOR in BT-549 and SK-BR-3 was determined using acyl-biotinyl exchange (ABE) assay. **D.** ABE analysis of mTOR palmitoylation in BrCa cells.** E.** mTOR palmitoylation was upregulated by ZDHHC22 overexpression detached by ABE assay. **F.** Palmitoylation sites of mTOR. **H.** BT-549 and SK-BR-3 cells were transfected with Vector and ZDHHC22 plasmids for 48 hours, followed by 200μg/ml cycloheximide (CHX) treatment for the indicated times. mTOR protein expression levels were detected with western blot and quantified with Image J software. Data are presented as the mean ± SEM. ***P < 0.001. HAM: hydroxylamine, ZDC22: ZDHHC22, ZDC22-mut: ZDHHC22-(C111A) mutation.

To explore whether mTOR is regulated by ZDHHC22, we first examined the interaction between ZDHHC22 and mTOR. The binding of ZDHHC22 and endogenous mTOR was demonstrated by co-immunoprecipitation assays in cells that stably overexpress ZDHHC22. The results revealed that ZDHHC22 is bound to mTOR and vice versa (Fig. 6A). The Immunofluorescence assay confirmed that ZDHHC22 mainly co-localized with mTOR (Fig. 5A). Moreover, ZDHHC22-mut (C111A) is also bound to mTOR (Fig. 6A and Fig. S2A), indicating that the C111A mutation affected the palmitoyltransferase activity, but not the binding affinity between ZDHHC22 and mTOR.

To further investigate how ZDHHC22 affected mTOR, we first identified mTOR was regulated by palmitoylation. After being treated with 25 μM 2-bromopalmitate (2-BP), a general palmitoylation inhibitor, mTOR expression was significantly elevated in BT-549 and SK-BR-3 cells, which was consistent with the increased p-AKT(Ser-473) (Fig. 6B). Next, we subjected BT-549 and SK-BR-3 cells to ABE assays to directly detect the protein palmitoylation levels of mTOR 33. Palmitoylation of mTOR was detected by western blot with streptavidin-HRP (Fig. 6C). As previously described, the expression of ZDHHC22 is increased in ER-positive BrCa cell lines like MCF-7 and T47D compared with the ER-negative BrCa cell lines such as BT-549 SK-BR-3 and YCC-B1. The palmitoylation level of mTOR was detected in these cell lines. As our expectation, a higher mTOR palmitoylation level was observed in the MCF-7 and T47D cell lines (Fig. 6D). In addition, the ABE assay results indicated that ZDHHC22 increased mTOR palmitoylation, and these effects were abolished by the C111A mutation (Fig. 6E).

According to the prediction of CSS-Plam 4.0 (http://www.csspalm.biocuckoo.org/), there are four possible palmitoylation sites, cysteine residues 361, 362, 1497, and 2133, respectively, within the mTOR protein. We identified cysteine 361 and 362 were the major palmitoylation sites in different species. Mutation of cysteine 361 and 362 to alanine (mTOR-mutation) was designed, and we found that mTOR palmitoylation was reduced (Fig 6G). Thus, we hypothesized that cysteine 361 and 362 contributed mostly to mTOR palmitoylation.

Since palmitoylation is an important post-translational modification that could regulate protein localization, stability, and protein-protein interactions 7, the half-life of mTOR was next determined by treatment with the protein synthesis inhibitor cycloheximide. Western blotting assays showed that ZDHHC22 overexpression, but not ZDHHC22-C111A mutation, significantly increased the turnover rate of mTOR in both BT-549 and SK-BR-3 cells (Fig. 6H and Fig S2C). Together, these results suggested that ZDHHC22 attenuated mTOR stability by improving its palmitoylation level.

### mTOR overexpression partially reverses the effects of ZDHHC22 on BrCa cell phenotypes

**Figure 7:**
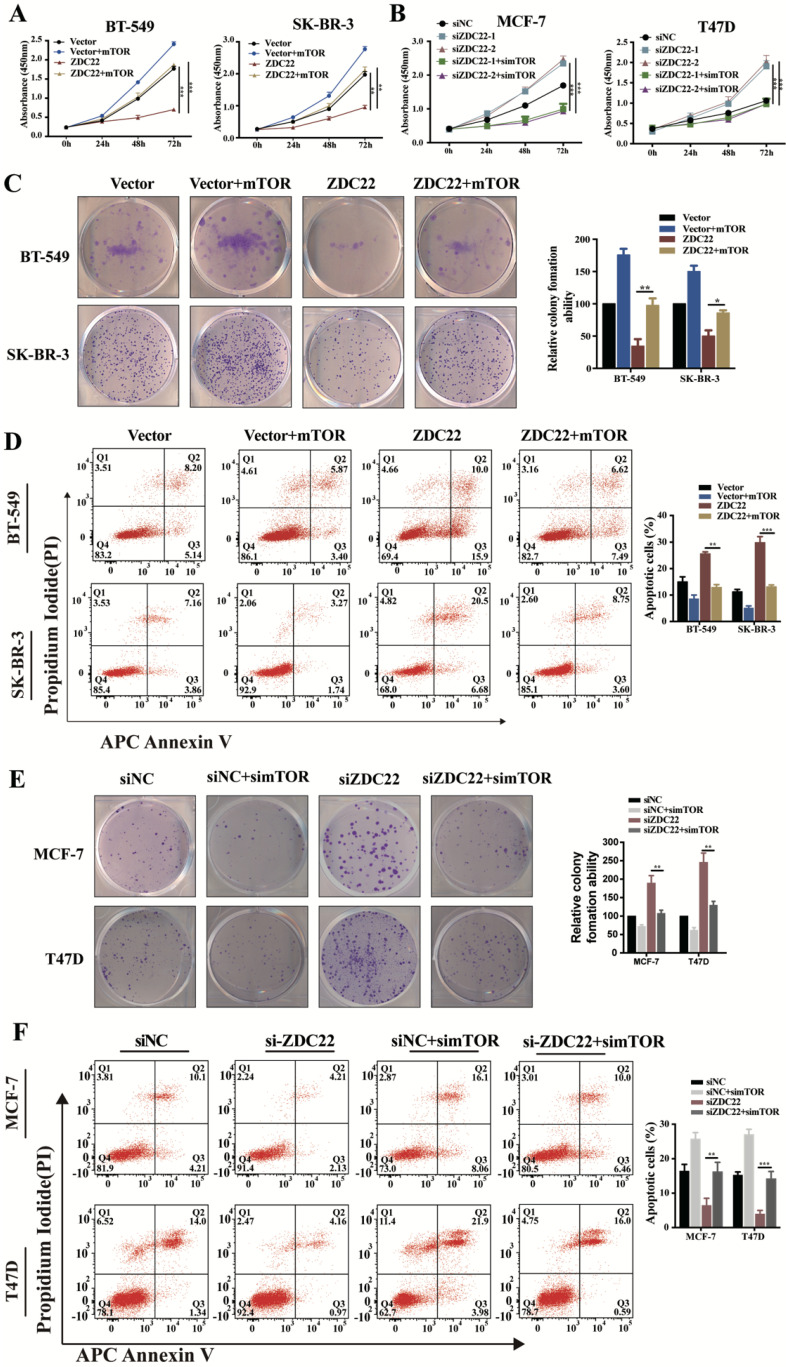
** mTOR reversed the tumor-inhibiting effects of ZDHHC22 in BrCa cells.** BT-549 and SK-BR-3 cells were transfected with mTOR or ZDHHC22 plasmid or co-transfected with two plasmids. Cell proliferation was determined by CCK8 assay (**A**) and colony formation assays (**C**), and the percentage of apoptotic cells was detected by flow cytometry (**D**). Effects of siZDHHC22-1/2 and simTOR on the proliferation and apoptosis of MCF-7 and T47D cells were evaluated by CCK8 assay (**B**), colony formation assays (**E**), and flow cytometry (**F**). Data are presented as the mean ± SEM of three independent experiments. **P < 0.01. ***P < 0.001. ZDC22:ZDHHC22, ZDC22-mut: ZDHHC22-(C111A) mutation.

To validate whether mTOR was involved in ZDHHC22-mediated BrCa cells proliferation, we overexpressed mTOR in ZDHHC22-overexpressing BT-549 and SK-BR-3 and, at the same time, knocked down mTOR expression in ZDHHC22-down expressing MCF-7 and T47D. We found that mTOR overexpression promoted proliferation while inhibiting apoptosis in ZDHHC22- overexpressing cells (Fig. 7A, C-D). Moreover, depletion of mTOR efficiently blocked the cell proliferation and promoted apoptosis in ZDHHC22 knockdown cells (Fig. 7B, E-F). Collectively, these results revealed that ZDHHC22 affected BrCa cell proliferation through the regulation of mTOR.

### ZDHHC22 restores sensitivity toward endocrine therapy

**Figure 8:**
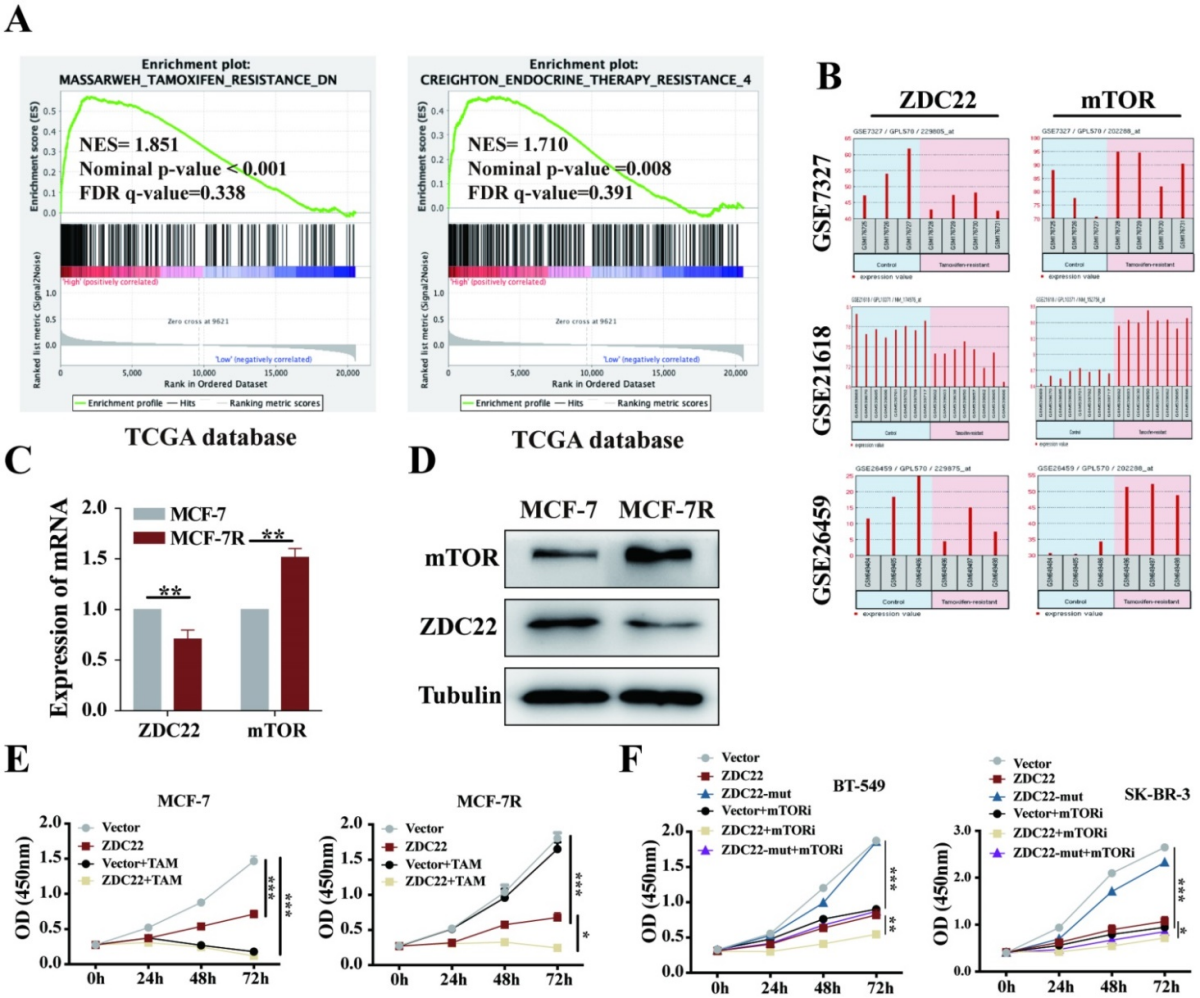
** The ZDHHC22 restores sensitivity toward tamoxifen therapy. A.** GSEA analysis detached ZDHHC22 was significantly associated with Tamoxifen and endocrine therapy resistance.** B.** The ZDHHC22 and mTOR mRNA expression in tamoxifen-resistant breast cancer cells based on microarray datasets GSE7327, GSE21618, and GSE26459. **C.** The mTOR and ZDHHC22 mRNA expression in tamoxifen-resistant MCF-7 cells (MCF-7R) and parental cells (MCF-7) using the qRT-PCR assay. **D.** The mTOR and ZDHHC22 protein expression in MCF-7R and MCF-7 cells using WB assay. **E.** MCF-7 and MCF-7R cells were transfected with Vector and ZDHHC22 plasmid for 48 hours, followed by 20 μM tamoxifen treatment, respectively. Cell growth was measured with a CCK-8 assay at the indicated time. **F.** BT-549 and SK-BR-3 cells were transfected with Vector, ZDHHC22 and ZDHHC22-(C111A) plasmids, followed by 10 μM KU-0063794 treatment, respectively. Cell growth was measured with a CCK-8 assay at the indicated time. Data are presented as the mean ± SEM of three independent experiments. *P < 0.05. **P < 0.01. ***P < 0.001. ZDC22:ZDHHC22.

The GSEA analysis using the TCGA database also showed that ZDHHC22 was associated with Tamoxifen and endocrine therapy resistance. As described above, ZDHHC22 was higher in ER-positive breast tumors and inversely associated with mTOR expression. The mTOR signal is often overactive in ER-positive breast tumors that resist endocrine therapy 42. Thus, we hypothesized that there might be a potential correlation between ZDHHC22 and BrCa endocrine resistance. Analysis of the GEO database (datasets GSE7327, GSE21618, and GSE26459) indicated that mTOR was always overexpressed, and at the same time, ZDHHC22 was frequently downregulated in tamoxifen-resistant BrCa cells compared to the control cells (Fig. 8B). Therefore, tamoxifen-resistant BrCa cell (MCF-7R) and parental cell (MCF-7) were used to detect the expression of mTOR and ZDHHC22. As shown, the mRNA and protein level of mTOR was significantly increased while the ZDHHC22 was significantly decreased in MCF-7R cells compared with MCF-7 cells (Fig. 8C-D). CCK-8 assay showed that overexpression of ZDHHC22 in MCF-7 cells suppressed cell proliferation but did not enhance the effect of tamoxifen treatment. However, ZDHHC22 overexpression significantly re-sensitized MCF-7R cells to tamoxifen treatment (Fig. 8E). These results seemed to elucidate a link between ZDHHC22, ER, and mTOR.

Besides, we evaluated the effects of ZDHHC22 combined with a mTOR kinase inhibitor. Cells were treated with KU-0063794, an ATP-competitive inhibitor of mTOR, which effectively inhibited mTORC2 and mTORC1 43. The CCK-8 results showed that the cell growth rate of the ZDHHC22 group was similar to that of the 10 μM KU-0063794 group. The combination of ZDHHC22 and KU-0063794 exhibited a synergistic reaction to cell growth inhibition (Fig. 8F).

### ZDHHC22 inhibits breast cancer cell growth *in vivo*

**Figure 9:**
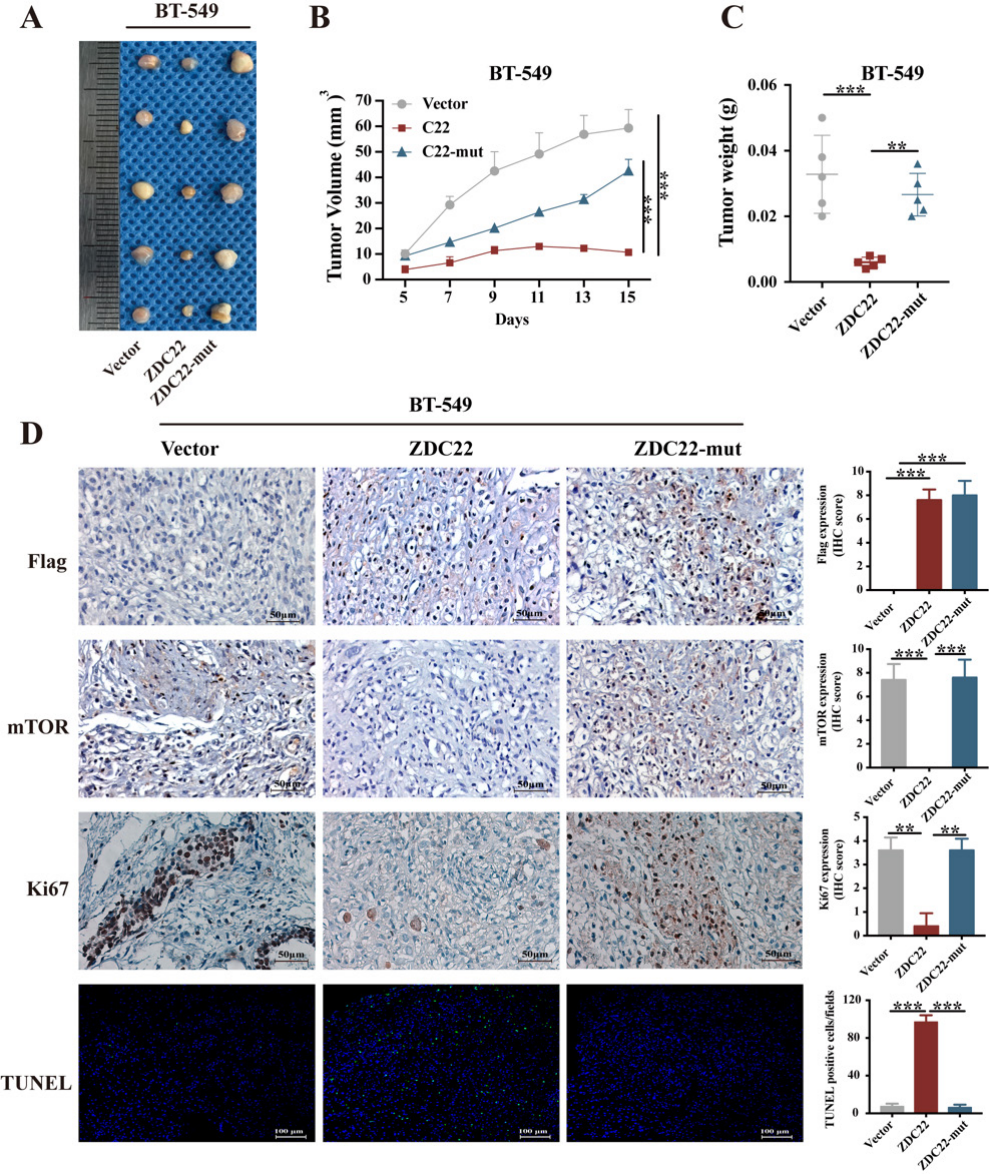
** Ectopic ZDHHC22 inhibits breast cancer proliferation *in vivo*, while ZDHHC22-(C111A) mutation abolished these effects.** BT-549 cells stably expressed with vector, ZDHHC22 and ZDHHC22 (C111A) were injected subcutaneously into the mammary fat pads of female nude mice (n = 5), respectively. Tumor sizes were measured every 2 days for 2 weeks. **A.** Image of xenografts after excised. **B.** Growth curve of xenograft tumors. **C.** Tumor weight of xenograft tumors. **D.** Representative images of IHC. Flag-ZDC22, mTOR and Ki-67 stained section of primary tumor tissues isolated from nude mice bearing BT-549 cells stably expressed with vector, ZDHHC22, and ZDHHC22-mut, respectively. Apoptotic cells in primary tumor tissues isolated from nude mice bearing BT-549 cells stably expressed with vector, ZDHHC22, and ZDHHC22-mut, respectively, were detected with TUNEL assay. Values represent mean ± SEM. **P < 0.01. ***P < 0.001. ZDC22:ZDHHC22, ZDC22-mut: ZDHHC22-(C111A) mutation.

The xenograft model of a nude mouse bearing BT-549 cells was established to investigate the effects of ZDHHC22 on tumor growth* in vivo*. Tumor growth in the ZDHHC22 group was remarkably slower than that in the control group and C111A group (Fig. 9A). The volume and weight of the harvested xenografts in different groups exhibited a similar trend (Fig. 9B-C). IHC staining revealed that tumors in the ZDHHC22 group had a lower percentage of Ki-67 positive cells and mTOR expression than tumors in the other two groups (Fig. 9D), indicating ectopic ZDHHC22 expression suppressed the proliferation and expression of mTOR protein of breast cancer. The TUNEL staining of the primary tumors showed that the proportion of apoptotic breast cancer cells was increased after ZDHHC22 overexpression (Fig. 9D). Together, these findings indicated that ZDHHC22 inhibits BrCa tumorigenesis *in vivo*.

## Discussion

As the main undertaker of palmitoylation, the ZDHHC family has important regulatory effects on various cancers, especially in their development and chemoresistance 5. In the present study, we found that the ZDHHC22 expression level was associated with ER status in BrCa due to hypermethylation of its promoter. Gain/loss-of-function experiments showed ZDHHC22 significantly inhibited the BrCa cell growth in vivo and in vitro. As a palmitoyltransferase, the anti-tumor effect of ZDHHC22 on BrCa depends on its enzyme activity. Mechanistic studies indicated that ZDHHC22 reduced mTOR protein stability by palmitoylation, inhibiting the activity of mTOR downstream substrates. Furthermore, ZDHHC22 expression was negatively correlated with mTOR in tamoxifen-resistant BrCa cells, and restoring ZDHHC22 expression could improve the sensitivity of Tamoxifen-resistant strains in BrCa endocrine therapy.

The PI3K/AKT/mTOR signaling pathway plays an important role in cancer cell growth, motility, and apoptosis 44, 45. This pathway is frequently activated during the development of different subtypes of BrCa 46, 47. AKT and mTOR, the two main nodes of this pathway, have become the key targets of tumor therapy 45, 48. Our study found that ZDHHC22 could inhibit the proliferation and promote apoptosis of BrCa by inhibiting the AKT/mTOR signaling pathway. SwissPalm and TermiNator online tools predict mTOR as the substrate of ZDHHC22. Thus, we hypothesized that ZDHHC22 dephosphorylates AKT through regulating mTOR (both mTORC2 and mTORC1 complexes). Immunofluorescence analysis also confirmed that ZDHHC22 is mainly co-located with mTOR in the plasma membrane and endoplasmic reticulum, the two main locations where ptdlns-3,4,5-p3 (PIP3) recruits AKT and is phosphorylated by mTORC2 38. The phosphorylation of mTORC1 and its downstream substrates S6K and 4E-BP1 were also reduced due to the overexpression of ZDHHC22, which illustrated that ZDHHC22 inhibited mTOR, including mTORC2 and mTORC1 complex.

Co-immunoprecipitation assays were performed to verify the interaction between ZDHHC22 and mTOR, and the binding between ZDHHC22 and endogenous mTOR was determined. Some studies predicted that mTOR might be regulated by palmitoylation 49, 50; however, to the best of our knowledge, the degree and regulatory site of mTOR palmitoylation has not been analyzed. The effects of ZDHHC22 on mTOR palmitoylation were validated by ABE assay; the results revealed that ZDHHC22 increased mTOR palmitoylation, and the C111A mutation abolished these effects. Furthermore, C361 and C362 sites contributed mainly to mTOR palmitoylation.

In addition, a large number of studies have shown that the activation of mTOR leads to drug resistance to most anti-cancer therapies 51, 52. Arteaga et al. reported that neratinib resistance in HER2-mutant cancers was closely correlated with mTOR activation 53. Peng et al. found that KRAS-mutant lung cancer cells resisted the chemotherapeutic drugs depending on the mTOR signal activation 54. Clinically, the mTOR inhibitor everolimus is often used to restore the sensitivity to endocrine therapy in advanced ER-positive, HER2-negative BrCa 42. Since both ZDHHC22 and mTOR significantly correlated with ER status, we next explore the possible relationship between ZDHHC22, mTOR, and endocrine therapy resistance. Our results illustrated that mTOR was overexpressed in MCF-7R cells, while ZDHHC22 was down-regulated in MCF-7R cells. Furthermore, ectopic ZDHHC22 expression can restore the sensitivity of MCF-7R cells to endocrine therapy. To further confirm the recovery function of ZDHHC22 against endocrine therapy resistance, clinical specimens and patient-derived xenografts may be a better experimental model, which needs further research.

## Conclusions

In conclusion, we have revealed the expression pattern and pathological function of ZDHHC22 in BrCa, ZDHHC22-mediated mTOR palmitoylation, and inhibited the AKT signaling pathway to decrease malignant proliferation and endocrine resistance of BrCa cells. These findings enrich the regulation role of the ZDHHC family in cancer development and progression and provide a theoretical basis for exploring specific palmitoylation drugs for endocrine therapy resistance of breast tumors in the future.

## Supplementary Material

Supplementary figures and tables.

## Figures and Tables

**Table 1:** Correlation of ZDHHC22 expression level with the clinicopathological features in breast cancer patients of TCGA database.

Characteristics	Number of cases	ZDHHC22		
		Low	High	*P*-value
Age				
≤60	594	307 (51.7%)	287 (48.3%)	0.196
>60	482	230 (47.7%)	252 (52.3%)	
Tumor size				
≤2cm	210	94 (44.8%)	116 (55.2%)	0.286
>2cm	581	285 (49.1%)	296 (50.9%)	
Lymph node metastasis				
Negative	385	189 (49.1%)	196 (50.9%)	0.519
Positive	406	190 (46.8%)	216 (53.2%)	
AJCC Stage				
I	133	63 (47.4%)	70 (52.6%)	0.222
II	446	223 (50.0%)	223 (50.0%)	
III	175	74 (42.3%)	101 (57.7%)	
ER				
Negative	179	112 (62.6%)	67 (37.4%)	<0.0001*
Positive	601	268 (44.6%)	333 (55.4%)	
PR				
Negative	255	151 (59.2%)	104 (40.8%)	<0.0001*
Positive	522	229 (43.9%)	293 (56.1%)	
HER2				
Negative	652	306 (46.9%)	346 (53.1%)	0.031*
Positive	114	66 (57.9%)	48 (42.1%)	
*p < 0.05 indicates statistical significance.				

**Table 2:** Correlation of ZDHHC22 methylated status with the clinicopathological features in breast cancer patients.

Characteristics	Numbers of cases	ZDHHC22		
		Methylated	Unmethylated	*P*-value
Age				0.881
≤60	42	18 (42.86%)	24 (57.14%)	
>60	22	9 (40.91%)	13 (59.09%)	
Tumor grade				0.116
1	3	3 (100%)	0 (0%)	
2	46	16 (34.78%)	30 (65.22%)	
3	2	1 (50.0%)	1 (50.0%)	
Unknown	13	7 (53.85%)	6 (46.15%)	
Tumor size				0.213
≤2cm	30	14 (46.67%)	16 (53.33%)	
>2cm	32	13 (40.63%)	19 (59.37%)	
Unknown	2	0 (0%)	2 (100%)	
Lymph Node Metastasis				0.104
Negative	28	15 (53.57%)	13 (46.43%)	
Positive	36	12 (33.33%)	24 (66.67%)	
ER status				0.035*
Negative	22	13 (50.0%)	13 (50.0%)	
Positive	35	12 (34.29%)	23 (65.71%)	
Unknown	7	2 (66.67%)	1 (33.33%)	
*P < 0.05 indicates statistical significance.				

## Abbreviations

ZDC22: ZDHHC22
ZDC22-mut: ZDHHC22-C111A mutation
BrCa: breast cancer
mTOR: the mammalian target of rapamycin
PKB/AKT: protein kinase B
RTK: receptor tyrosine kinase
MCF-7R: tamoxifen-resistant MCF-7
TCGA: The Cancer Genome Atlas
GEO: Gene Expression Omnibus database
GSEA: Gene set enrichment analysis
qPCR: quantitative reverse transcription PCR
MSP: methylation-specific PCR
RT-PCR: Semiquantitative PCR
A+T: 5-Aza-2′- deoxycytidine and trichostatin A (TSA)
OD: optical density value
ABE: acyl-biotin exchange assay
IHC: immunohistochemistry staining
DAPI: 4'-6-diamidino-2-phenylindole
PI: propidium iodide
M: methylated
U: unmethylated
