# Supplementary material for: ZDHHC22-mediated mTOR palmitoylation restrains breast cancer growth and endocrine therapy resistance
Source: Int J Biol Sci. 2022 Apr 4;18(7):2833–50. doi: 10.7150/ijbs.70544 (PMC9066102; doi:10.7150/ijbs.70544)
Supplement: Supplementary file 1 — Supplementary figures and tables. [file ijbsv18p2833s1.pdf]

SUPPLEMENTAL FIGURES :

FIGURE S1. A. Promoter methylation of ZDHHC22 in 64 breast cancer tissue samples. **B.** Validation of ZDHHC22 and ZDHHC22-(C111A) mRNA and protein expression by qPCR and western blot analyses. **C-D.** Cell vitality was detected with CCK-8 assay and colony formation assay after transfection with Vector, ZDHHC22 and ZDHHC22-(C111A) plasmids in YCC-B1 cells. **E-F.** Cell cycle and cell apoptosis in YCC-B1 cells at 48 hours post-transfection of Vector, ZDHHC22 and ZDHHC22-(C111A) plasmids was examined by flow cytometry analysis of PI staining and Annexin V-APC/PI staining, respectively. **G** Immunofluorescence assays were used to detect the protein expression of PHLPP2 after transfected with ZDHHC22 plasmids. Values represent mean ± SD. from three independent experiments, $^{**}p < 0.01$. $^{***}p < 0.001$. ZDC22: ZDHHC22, ZDC22-mut: ZDHHC22-(C11A) mutation.

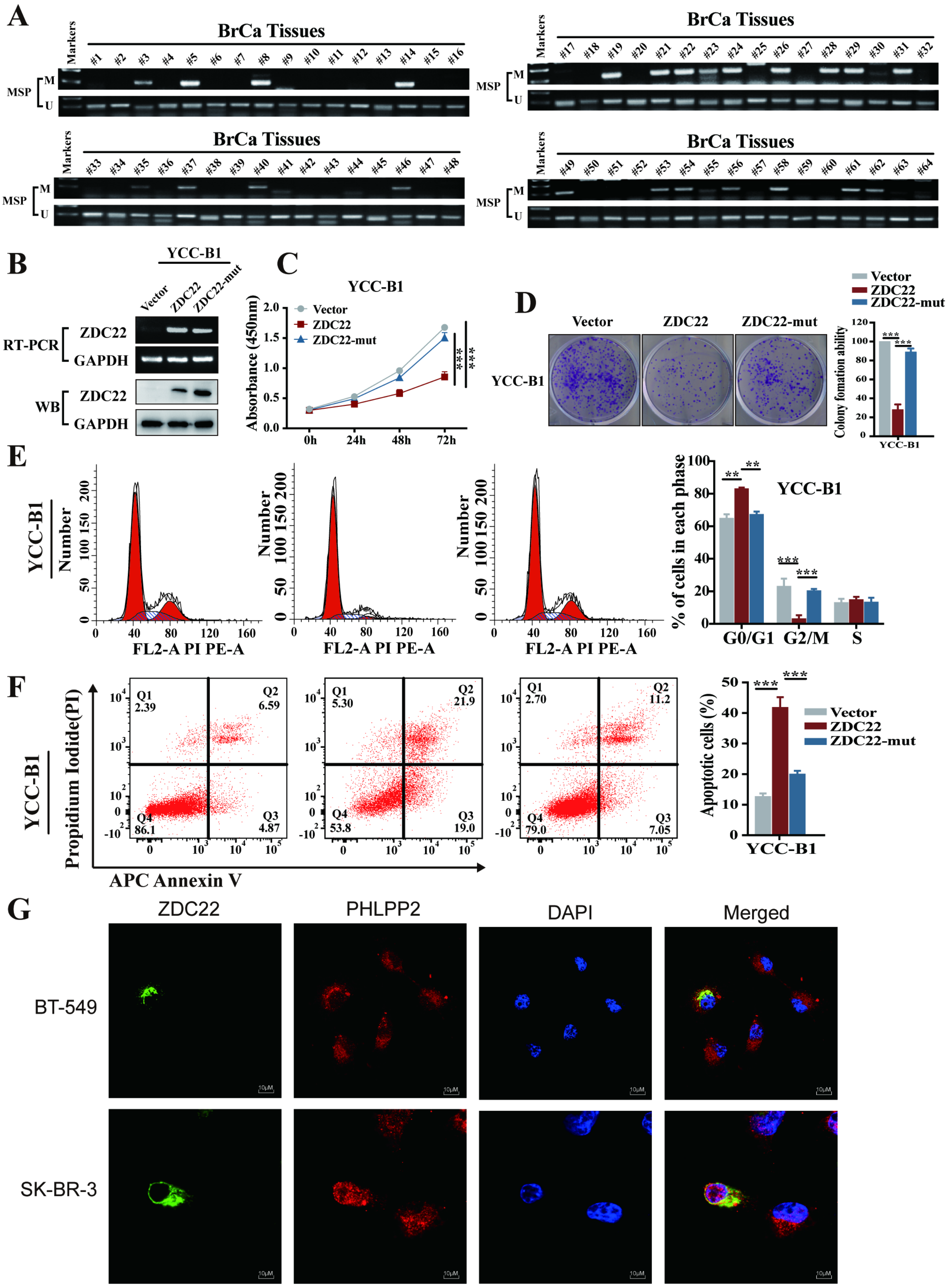
A
BrCa Tissues
Markers
MSP
M
U
#1 #2 #3 #4 #5 #6 #7 #8 #9 #10 #11 #12 #13 #14 #15 #16
#17 #18 #19 #20 #21 #22 #23 #24 #25 #26 #27 #28 #29 #30 #31 #32
#33 #34 #35 #36 #37 #38 #39 #40 #41 #42 #43 #44 #45 #46 #47 #48
#49 #50 #51 #52 #53 #54 #55 #56 #57 #58 #59 #60 #61 #62 #63 #64
B
YCC-B1
Vector
ZDC22
ZDC22-mut
RT-PCR
WB
ZDC22
GAPDH
C
YCC-B1
Vector
ZDC22
ZDC22-mut
Absorbance (450nm)
0h
24h
48h
72h
***
***
D
Vector
ZDC22
ZDC22-mut
YCC-B1
Colony fomation ability
***
***
E
YCC-B1
Number
FL2-A PI PE-A
% of cells in each phase
G0/G1
G2/M
S
**
**
***
***
F
YCC-B1
Propidium Iodide(PI)
APC Annexin V
Q1 2.39
Q2 6.59
Q3 4.87
Q4 86.1
Q1 5.30
Q2 21.9
Q3 19.0
Q4 53.8
Q1 2.70
Q2 11.2
Q3 7.05
Q4 79.0
Apoptotic cells (%)
Vector
ZDC22
ZDC22-mut
***
***
G
ZDC22
PHLPP2
DAPI
Merged
BT-549
SK-BR-3
10μM

FIGURE S2. A. Immunofluorescence assays were used to detect the protein expression and co-localization of mTOR at the indicated time in BT-549 and SK-BR-3 cells transfected with ZDHHC22-(C11A) plasmids. **B.** The ZDHHC22 mainly co-localized with the endoplasmic reticulum. **C.** BT-549 and SK-BR-3 cells were transfected with Vector and ZDHHC22-mut plasmids for 48 hours, followed by 200μg/ml cyclohexmide (CHX) treatment for the indicated times. mTOR protein expression levels were detected with western blot and quantified with Image J software. ZDC22:ZDHHC22, ZDC22-mut: ZDHHC22-(C11A) mutation.

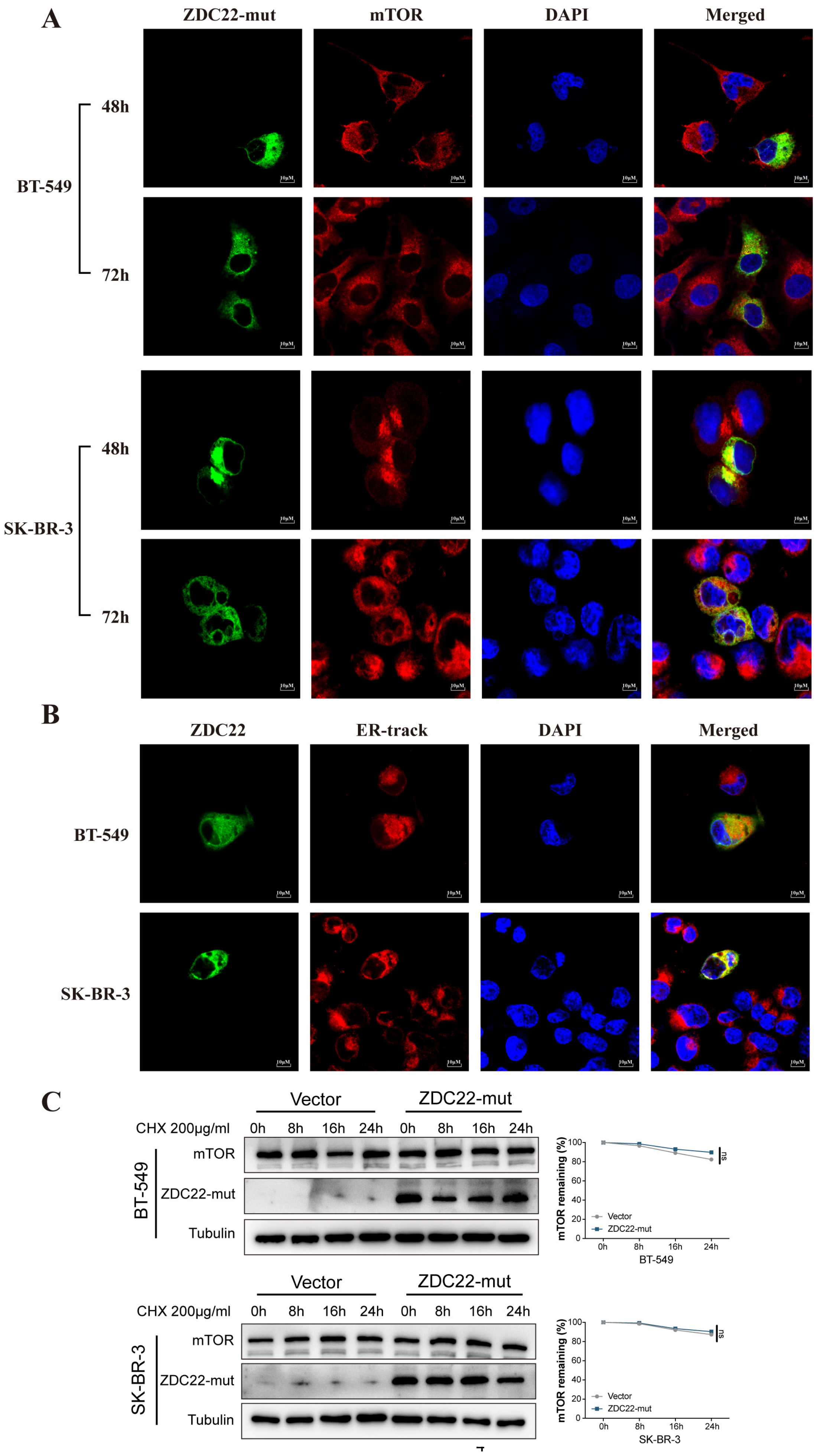

A
ZDC22-mut
mTOR
DAPI
Merged
BT-549
48h
72h
SK-BR-3
48h
72h
B
ZDC22
ER-track
DAPI
Merged
BT-549
SK-BR-3
C
Vector
ZDC22-mut
CHX 200μg/ml
0h
8h
16h
24h
BT-549
mTOR
ZDC22-mut
Tubulin
mTOR remaining (%)
Vector
ZDC22-mut
ns
SK-BR-3

1

TABLE S1. List of expression primers used in this study.

**PCR**	**Primer**	**Sequence (5'-3')**	**Product size**	**PCR Cycles**
RT-PCR	ZDHHC22F	GTGACCTTCGTGCTGCAGCT	167bp	35
	ZDHHC22R	AGGTCGTCTGGGGAGTTCTG		35
	GAPDHF	GGAGTCAACGGATTTGGT	206bp	23
	GAPDHR	GTGATGGGATTTCCATTGAT		23
qRT-PCR	mTORF	TGTGGGCAGCATCACTCTT	80bp	
	mTORR	GGCGAACAAATTGGGTCAG		
	GAPDHF	CCAGCAAGAGCACAAGAGGAA	114bp	
	GAPDHR	CAAGGGGTCTACATGGCAACT		

Note: RT-PCR: Semiquantitative reverse transcription PCR;
qRT-PCR: quantitative real-time PCR.

TABLE S2. List of MSP primers used in this study.

MSP	Primer	Sequence (5'-3')
ZDHHC22m1	ATAAGAGGAGTTTCGGACGTC	101bp
ZDHHC22m2	CCAATTCCCGAAACGAAACG	
ZDHHC22u1	GATAAGAGGAGTTTTGGATGTT	104bp
ZDHHC22u2	ACCCAATTCCCAAAACAAAACA	

Note: MSP: methylation-specific PCR

TABLE S3. Prediction of mTORC2 complex and AKT palmitoylation

Protein	N-terminal sequence or entry code	Predicted N-terminus of the mature protein	Likelihood (%)
mTOR	NP_004949.1	*M (1)	100
Deptor-X1	NP_073620.2	Ac-M (1)	100
Deptor-X2	NP_001269941.1	Ac-M (1)	100
Protor1-X1	NP_851850.1	M (1)	89
Protor1-X2	NP_001017528.1	AC-S (2)	85
Protor1-X3	NP_00107529.1	V (2)	100
Protor1-X4	NP_001185650.1	V (2)	100
Sin1-X1	NP_001006618.1	Ac-A (2)	83
Sin1-X2	NP_007022.1	Ac-A (2)	83
Sin1-X3	NP_001006620.1	Ac-A (2)	83
Sin1-X4	NP_001006621.1	Ac-T (2)	77
Sin1-X5	NP_001006619.1	Ac-A (2)	83
mLST8-Xa	NP_001186102.1	Ac-M (1)	67
mLST8-Xb	NP_001186104.1	Ac-M (1)	67
mLST8-Xc	NP_001338986.1	Ac-M (1)	67
mLST8-Xd	NP_001338988.1	M (1)	89
Rictor-X1	NP_689969.2	Ac-A (2)	83
Rictor-X2	NP_001272368.1	Ac-A (2)	83
Rictor-X3	NP_001272369.1	My-G (2)	45
AKT-X1	NP_001014431.1	AC-S (2)	85
AKT-X2	NP_001014432.1	AC-S (2)	85
AKT-X3	NP_005154.2	AC-S (2)	85
